# SIRT1 metabolic actions: Integrating recent advances from mouse models^★^

**DOI:** 10.1016/j.molmet.2013.10.006

**Published:** 2013-10-23

**Authors:** Marie Boutant, Carles Cantó

**Affiliations:** Nestlé Institute of Health Sciences S.A., EPFL campus, Quartier de l’Innovation, Bâtiment G, CH-1015 Lausanne, Switzerland

**Keywords:** SIRT1, Transgenic models, Energy homeostasis, Insulin resistance

## Abstract

SIRT1 has attracted a lot of interest since it was discovered as a mammalian homolog of Sir2, a protein that influences longevity in yeast. Intensive early research suggested a key role of SIRT1 in mammalian development, metabolic flexibility and oxidative metabolism. However, it is the growing body of transgenic models that are allowing us to clearly define the true range of SIRT1 actions. In this review we aim to summarize the most recent lessons that transgenic animal models have taught us about the role of SIRT1 in mammalian metabolic homeostasis and lifespan.

## 1 Introduction

SIRT1 (EC=3.5.1.-) is a NAD^+^-dependent protein deacetylase and the best studied mammalian homolog of the yeast enzyme Sir2, a protein with an established capacity to influence yeast replicative lifespan [1]. Consequently, the initial interest in SIRT1 spread rapidly due to its possible role in eukaryote lifespan regulation. While the role of SIRT1 in mammalian lifespan is still a matter of debate [2], we will review below the evidence from animal models suggesting that SIRT1 plays key roles in metabolic regulation and adaptation. This, in turn, impinges on the likelihood/tendency of organisms to develop metabolic and age-related diseases, including insulin resistance, cancer and neurodegeneration.

## 2 SIRT1 whole body gain- or loss-of-function

Despite conflicting results on whether SIRT1 homologs truly enhance longevity in lower eukaryotes [2], it seems clear that SIRT1 overexpression does not enhance maximal lifespan in mice under regular food regimes [3]. However, SIRT1 transgenic mice are protected against the metabolic damage induced by high-fat diets (HFD) [4,5]. For the moment, whether whole-body SIRT1 overexpression can also prevent the shortening of lifespan generally observed upon high-fat feeding [6,7] remains to be explored.

The first SIRT1 gain-of function model displayed several features resembling calorie restriction (CR): the transgenic mice were leaner, metabolically more active, and had improved glucose tolerance [8]. Two additional SIRT1 transgenic lines were later generated. Both of them concluded that mild overexpression of SIRT1 (2- to 4-fold higher, depending on the tissue) prevented HFD-induced glucose intolerance, insulin resistance and hepatic steatosis, despite no significant alteration in body weight [4,5]. More recent efforts have also shown that mice with higher global SIRT1 overexpression have enhanced mitochondrial content and display greater mitochondrial function in skeletal muscle [9]. This feature could provide an interesting mechanism to explain the protective metabolic phenotypes observed. However, the link between SIRT1 gain-of-function and mitochondrial biogenesis is not clear *in vivo*, as no enhanced mitochondrial gene expression was observed in the skeletal muscle of other whole-body SIRT1 transgenic lines [4,10]. One of the major caveats of the gain-of-function approaches is that higher SIRT1 expression does not necessarily result in increased SIRT1 activity. This has been demonstrated recently in models of aging, where reduced availability of NAD^+^ (the essential cosubstrate for the sirtuin catalytic reaction) compromises SIRT1 activity, despite higher SIRT1 content [11].

The presented observations have fueled an interest in understanding how the deletion of SIRT1 impacts global metabolic homeostasis. This has proven a nontrivial task. Whole-body deletion of SIRT1 leads to elevated prenatal death in inbred mice [12,13]. The few pups born displayed marked cardiac and neurological problems, leading to death very early in the postnatal period [12,13]. In order to bypass this situation, SIRT1 deletion was performed in outbred mice. These mice were viable, but smaller, and displayed a marked metabolic inefficiency [14]. SIRT1 deficient mice were lethargic, ate more and had higher oxygen consumption [13]. This metabolic inefficiency impaired their ability to metabolically adapt to CR [14]. Recently, an elegant model has been developed in order to genetically ablate SIRT1 exclusively in adulthood [9]. The deletion of SIRT1 in adult mice did not result in any overt phenotype. Similarly, there were no obvious differences between SIRT1 knock-out (KO) and wild-type (WT) mice on most metabolic parameters, although weight gain was slightly lower in the KOs when placed on a HFD [9]. Another model worth discussing is the SIRT1 heterozygous mouse. Heterozygous SIRT1 KO mice were normal in body weight, fat content, and lean body mass relative to their WT littermates [15]. Accordingly, they did not display any remarkable difference in a series of histologic and gene expression analyses. However, when placed on HFD these mice were more prone to develop hepatic steatosis and metabolic damage [15,16]. Overall, these models provide conclusive evidence that SIRT1 deletion leads to inefficient metabolism. While this does not manifest into an overt phenotype when fed regular diets, it renders mice more prone to metabolic complications upon dietary challenges. Conversely, whole body SIRT1 overexpression confers protection against HFD-induced insulin resistance [4,5], mostly by maintaining the ability of insulin to block hepatic glucose production [5]. In order to gain knowledge on the role of SIRT1 in particular tissues and their contribution to metabolic impairment, several tissue-specific SIRT1 deficient mouse models have been generated.

## 3 Liver-specific SIRT1 models

The liver plays a central role in the maintenance of blood glucose levels in the fasting state as well as in the regulation of cholesterol and lipid homeostasis. Early hypotheses proposed that SIRT1 could potentiate gluconeogenesis by directly deacetylating and enhancing the transcriptional activity of the Peroxisome Proliferator-activated Receptor (PPAR) gamma coactivator 1α (PGC-1α) or the Forkhead O-box protein 1 (FoxO1) transcription factor, both considered key positive controllers of the gluconeogenic transcriptional program [1]. To date, a number of independent liver-specific SIRT1 KO mice have been generated. Surprisingly, none displayed reduced fasting glycemia [17–20]. In fact, one model exhibited a tendency towards hyperglycemia even on chow diet with elevated glucose production upon fasting [17]. This is in stark contrast to earlier work using adenoviral-mediated hepatic SIRT1 overexpression which suggested that higher SIRT1 levels should promote hyperglycemia [21]. These results, however, have been recently challenged by findings indicating that liver overexpression of SIRT1 ameliorates hyperglycemia in insulin resistant mouse models [22]. Similarly, mice with global overexpression of SIRT1 do not show signs of hyperglycemia and are protected against glucose intolerance when fed high caloric diets [4,5]. Altogether, these observations indicate that SIRT1 is not in itself linked to enhanced hepatic glucose production, even if in some scenarios this might be the case. Rather, the transgenic mouse models suggest that SIRT1 activation generally leads to an attenuation of the gluconeogenic rate. Indeed, detailed time-course analyses during the fasting period revealed that SIRT1 activation occurs during rather late phases (24 h), and that SIRT1 activation leads to the deacetylation and degradation of the cAMP response element binding protein (CREB) regulated transcription coactivator 2 (CRTC2), which attenuates gluconeogenesis to a sustainable rate for extended periods [23]. Given the powerful ability of SIRT1 to enhance PGC-1α activity [24] and of PGC-1α to enhance gluconeogenesis [25,26], how come SIRT1 gain-of-function models do not show enhanced gluconeogenic rates? It might be that PGC-1α actions on gluconeogenic gene expression are also tightly regulated. PGC-1α regulation in the liver does not always correlate with gluconeogenic gene expression [27]. Similarly, activation of the AMP-activated protein kinase (AMPK) enhanced PGC-1α deacetylation in the liver, but blocked simultaneously gluconeogenic gene expression (Canto C and Johan Auwerx, unpublished observations [28]). Therefore, physiological increases in PGC-1α activity in the liver might not necessarily be linked to the induction of gluconeogenic gene expression, even if PGC-1α harbors this potential when combined with certain stimuli (e.g: cAMP signaling [25]) or when bound to specific transcription factors, such as FOXO1 [29] or HNF-4α [26,30].

Despite the apparent confusion regarding the impact of SIRT1 on liver glucose metabolism, most studies broadly agree that SIRT1 activation enhances oxidative metabolism in liver. The knock-down or genetic ablation of SIRT1 in liver induces hepatic lipid accumulation by upregulating the expression of lipogenic genes and reducing fatty acid oxidation [16,19–21]. This renders SIRT1 deficient livers more sensitive to HFD-induced hepatic steatosis [19]. Conversely, SIRT1 overexpressing mice are protected against hepatic lipid accumulation and inflammation when fed a HFD [22]. Strikingly, one of the liver-specific KO models displayed the unusual feature of being protected against hepatic steatosis [18]. The reasons for such a discordant observation in this model are not yet clear, but might arise from the different mixed background strains used in these reports.

At the molecular level, SIRT1 might enhance oxidative metabolism and prevent hepatic lipid accumulation through the activation of PGC-1α [21]. This is not surprising, as PGC-1α is a key downstream deacetylation target of SIRT1 in the regulation of mitochondrial and fatty acid oxidation gene expression. At the same time, SIRT1 positively controls fatty acid oxidation though PPARα activation [19]. The nuclear receptor PPARα regulates lipid metabolism, and more particularly, gene expression implicated in β-oxidation. In SIRT1 deficient livers, PPARα agonists fail to promote the expression of PPARα target gene [19]. Mechanistically, it was elegantly demonstrated that SIRT1 binds to the ligand- and DNA-binding domains of PPARα. Consistently, SIRT1 was found to be present on the promoter of PPARα-target genes [19]. Colocalisation with SIRT1 facilitates the efficient deacetylation of PGC-1α, which can then coactivate PPARα. In the absence of SIRT1, PGC-1α remains associated in a constitutively hyperacetylated state, which dampens PGC-1α coactivating activity [24] and blunts PPARα transcriptional activation. Conversely, SIRT1 inhibits lipogenic gene expression by acting as a negative regulator of the Sterol Regulatory Element Binding Protein (SREBP)-1c [21,31,32]. SREBP-1c is a transcription factor that promotes the expression of lipogenic and cholesterogenic genes in order to facilitate fat storage. The deacetylation of SREBP-1c by SIRT1 renders the protein susceptible to ubiquitin-mediated degradation [31]. Hence, SIRT1 activation leads to decreased SREBP-1c protein levels. This results in decreased occupancy of SREBP-1c on the promoter of lipogenic genes and a concomitant reduction in their expression levels [31,32].

**Figure 1:**
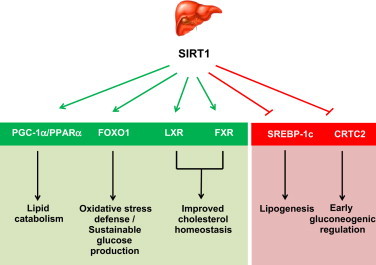
Schematic representation of the actions of SIRT1 in liver, based on genetically engineered mouse models. Through direct deacetylation, SIRT1 enhances the transcriptional activity of a series of regulators, such as PGC-1α, the FOXO family of transcription factors, and the Liver X Receptor (LXR) or the Farnesoid X receptor (FXR). However, a series of other transcriptional regulators, such as SREBP-1c and CRTC2 are downregulated through deacetylation by SIRT1. Overall, SIRT1 activation favors lipid catabolism *vs.* anabolism, leading to protection against oxidative stress and sustainable glucose production during prolonged fasting.

Finally, the transgenic mouse models also support a role for SIRT1 in cholesterol homeostasis. SIRT1 liver-specific KO mice display decreased hepatic expression of genes involved in reverse cholesterol transport [33]. This is consistent with the reduction of blood cholesterol levels in SIRT1 overexpressing livers [8,21]. Indeed, SIRT1 has been shown to modulate cholesterol metabolism *in vivo* though positive regulation of the Farnesoid X receptor (FXR) and the Liver X receptors (LRX), LXRα and LXRβ [33,34]. In the case of FXR, SIRT1 can directly deacetylate Lys^157^ and Lys^217^ [34]. Down regulation of hepatic SIRT1 increases FXR acetylation, which inhibits its heterodimerisation with the Retinoid X receptor (RXR)α and therefore, its transcriptional activity [34]. Hence, SIRT1 deletion in liver is sufficient to downregulate FXR-related transcriptional programs and lead to the formation of cholesterol gallstones [35]. As for LXR, ligand binding promotes the interaction with SIRT1 and subsequent deacetylation on Lys^432^ (LXR*α*) and on Lys^433^ (LXRβ), promoting their activation [33]. As a result, LXR targets are only partially activated in SIRT1 liver-specific KO mice on HFD, a condition where LXRs are highly active [18]. Accordingly, the knock-down of SIRT1 in liver also leads to decreased expression of CYP7A1, a *bona-fide* LXR target [21]. It is worth mentioning that LXRs are also a potent inducer of lipid anabolism by increasing SREBP-1c activity [36]. However, SIRT1 can deacetylate SREBP-1c resulting in proteasomal degradation [31]. Therefore, SIRT1 activation might promote the beneficial effects of LXR activity on cholesterol homeostasis while preventing the detrimental effects on lipid anabolism by deacetylating SREBP-1c. Altogether, most approaches indicate that SIRT1 overexpression improves cholesterol metabolism and prevents hepatic steatosis, while SIRT1 deletion in the liver favors lipid accumulation (Figure 1). In light of the phenotypes of the liver-specific SIRT1 KO mice, it was surprising to find that whole body SIRT1 overexpressing mice developed worse lipid profiles and larger atherosclerotic lesions than WT counterparts when placed on an atherogenic diet [37]. While it is puzzling that this was not observed in any of the above described models, it should draw attention to potential contraindications for the therapeutic use of SIRT1 activators under certain dietary conditions.

## 4 SIRT1 and skeletal muscle metabolism

### 4.1 SIRT1 and skeletal muscle development

Initial clues to the possible roles of SIRT1 in skeletal muscle were obtained when SIRT1 was identified as a negative myogenic regulator. Overexpression of SIRT1 impaired myotube formation while decreased SIRT1 triggered premature differentiation [38]. Mechanistically, this effect was explained through the ability of SIRT1 to repress the muscle transcriptional regulator MyoD, a critical determinant of skeletal muscle differentiation [38]. SIRT1 is also a key mediator by which nutrient restriction impairs muscle differentiation. Glucose starvation promotes the activation of AMPK which results in increased expression of nicotinamide phosphoribosyltransferase (Nampt) and supply of NAD^+^ to support SIRT1 activity [39]. However, while SIRT1 can act as a myogenic regulator in cultured myocytes, this does not appear to be relevant *in vivo*, as mice overexpressing or lacking SIRT1 in skeletal muscle do not display any overt muscle developmental phenotype compared to wild-type mice [40–42].

### 4.2 SIRT1 and mitochondrial biogenesis

SIRT1 has been described many times as a key regulator of mitochondrial biogenesis through the deacetylation of PGC-1α (for review, see [43]). For example, PGC-1α becomes deacetylated in skeletal muscle during fasting [44]. In cultured C2C12 cells, SIRT1 activation is required for PGC-1α-mediated induction of mitochondrial and fatty acid oxidation gene expression in response to nutrient depletion [44]. Similarly, PGC-1α becomes deacetylated after a bout of exercise [45]. AMPK is a master regulator of mitochondrial biogenesis [28] and seems to play a key role in triggering SIRT1 activity during energy stress in skeletal muscle. Indeed, the activation of SIRT1 in response to nutrient or energy deprivation depends on AMPK activation, both *in vitro* [39,46] and *in vivo* [46]. The link between AMPK and SIRT1 activities can be at least partially explained by two complementary mechanisms. Firstly, AMPK activation enhances NAD^+^ availability both in cultured myocytes [39,45] and skeletal muscle [45]. The increase in fat oxidation rates induced by AMPK permits an increase in NAD^+^ that is sufficient to activate SIRT1 in a relatively short time frame [45]. Additionally, AMPK triggers Nampt expression, which helps maintain a more sustained increase in NAD^+^ [39,45]. As an alternative possibility, it has been recently proposed that AMPK might phosphorylate SIRT1, disrupting the interaction with its negative regulator, Deleted in Breast cancer 1 (DBC1) [47]. However, the direct phosphorylation of SIRT1 by AMPK has not been observed previously by other labs [45,48], and the residues reported are poorly conserved, with poor adherence to the AMPK consensus motif. In an interesting twist, SIRT1 has also been proposed to modulate AMPK activity. Silencing SIRT1 prevented the activation of AMPK by diverse polyphenols in HepG2 cells [49]. Conversely, SIRT1 overexpression enhanced AMPK activity both in HepG2 cells and liver [49]. It was, hence, exciting to see that LKB1, the main upstream kinase for AMPK activation in skeletal muscle [50], was an acetylated protein in cultured cells and rodent tissues [51]. Acetylated LKB1 failed to shuttle from the nucleus to the cytoplasm. Upon deacetylation by SIRT1, LKB1 shuttled more efficiently to the cytoplasm, binding STRAD and MO25 and forming this way an active kinase complex [51]. Consequently, SIRT1 could potentially modulate AMPK activity by influencing the activity of LKB1. Recent evidence in C2C12 myotubes, primary hepatocytes and primary myoblasts supported this hypothesis by demonstrating that resveratrol and AICAR failed to stimulate AMPK in when SIRT1 was reduced by genetic knock-down or ablation [9]. Several observations, however, still result controversial, at least in the context of muscle physiology. First, slight modulations of LKB1 activity are unlikely to be limiting for AMPK activation: a ~50% reduction in LKB1 activity in skeletal muscle does not affect basal or AICAR-stimulated AMPK activity, and even hypomorphic mice with 10-fold lower LKB1 activity retain a significant ability to activate AMPK [50]. Second, specific SIRT1 deletion or overexpression in skeletal muscle does not lead to differences in basal AMPK activity or in its potential activation by exercise, AICAR or dietary restriction [40,42,52]. Finally, other labs have reported an intact ability of resveratrol or AICAR to stimulate AMPK in MEFs or in C2C12 myotubes defective for SIRT1 activity [45,53]. Overall, the above observations testify for a complex relationship between AMPK and SIRT1, where both might influence each other activities and where particular aspects of this co-regulation might depend on the tissue examined and the dosing of the pharmacological activator used [9,54,55].

**Figure 2:**
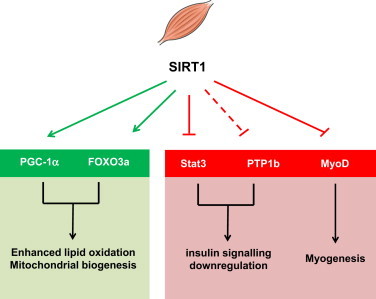
Schematic representation of the actions of SIRT1 in skeletal muscle, based on genetically engineered mouse models. SIRT1 activation in muscle favours lipid catabolism through the activation of lipid oxidation and mitochondrial biogenesis gene sets by PGC-1α and the FOXO family of transcription factors, mostly FOXO3a. SIRT1 also enhances insulin action through the repression of proteins that downregulate insulin signaling, either through direct deacetylation, as with STAT3, or by transcriptional means (dashed line), as with PTP1b. SIRT1 might also impact on muscle differentiation through the repression of MyoD, even though this has not been properly tested *in vivo*.

Considering the spectrum of substrates described for SIRT1 in skeletal muscle (Figure 2), one would expect that SIRT1 transgenesis would increase mitochondrial biogenesis in skeletal muscle. In line with this, global SIRT1 transgenic mice, with significant skeletal muscle overexpression of SIRT1, have been shown to display higher mitochondrial content [9]. This, however, contrasts with the lack of higher mitochondrial-related gene expression observed upon moderate SIRT1 overexpression [4,10]. Similarly, forced overexpression of SIRT1 through electroporation methods did not lead to increased mitochondrial content in rat muscle [56]. Interestingly, muscle-specific SIRT1 overexpressing mice have recently been generated [40]. Despite displaying >100-fold higher SIRT1 levels in skeletal muscle, these animals do not show any major phenotype at the level of energy expenditure or fat oxidation [40]. While mitochondrial content does not necessarily affect basal energy expenditure or fat oxidation rates, it will be important to evaluate muscle mitochondrial content in the muscle-specific SIRT1 overexpressing mice in order to judge whether the increases observed in Price et al. [9] are due to a muscle-autonomous function of SIRT1 or not.

Supporting a role for SIRT1 in muscle mitochondrial biogenesis, global deletion of SIRT1 in adulthood led to markedly impaired mitochondrial function [9]. In line with this, muscle-specific ablation of SIRT1 leads to a slight impairment in mitochondrial function [41], even though this was not clearly observed in another study using a similar mouse model [52]. The reason for this discrepancy might arise from the different Cre lines used to ablate the *Sirt1* gene in muscle. In both mouse models, the deletion of SIRT1 in skeletal muscle, however, did not have a major effect on energy metabolism of mice on regular diet [41,42]. Altogether, the muscle-specific mouse models indicate that the overexpression or deletion of SIRT1 does not have any major impact on muscle function in young, lean mice on the basal state.

### 4.3 SIRT1 and muscle insulin sensitivity

SIRT1 gain-of-function has been associated to higher glucose tolerance and insulin sensitivity [4,5], but, what is the contribution of skeletal muscle to this effect? Most evidence to date indicates that the contribution of muscle to this effect might be negligible. Muscle-specific overexpression of SIRT1 did not lead to enhanced insulin sensitivity [40]. This indicates that the enhancement of insulin sensitivity observed in whole-body SIRT1 overexpressing mice might stem from effects on other key tissues determining insulin sensitivity. In line with this, clamp studies indicate that the improvement in glucose homeostasis seen in SIRT1 transgenic mice on HFD are mostly due to increased hepatic insulin sensitivity rather than an increase in insulin-stimulated glucose disposal [5]. A key element to take into account, however, is that the higher insulin sensitivity reported for SIRT1 transgenic mice has been shown in mice on HFD [4,5], not on young, lean mice, as tested on the SIRT1 muscle-specific overexpressing mice [40]. However, supporting a lack of major effect of SIRT1 in skeletal muscle insulin sensitivity, the genetic ablation of SIRT1 in skeletal muscle did not affect whole body glucose disposal rates or insulin-stimulated glucose uptake in muscle in mice fed *ad libitum* [42]. Altogether, the evidence obtained in mouse models does not seem to indicate that skeletal muscle SIRT1 has an influence on insulin sensitivity and whole body glucose tolerance.

### 4.4 SIRT1 and the metabolic adaptations to nutrient/energy stress

A second concept originating from cell-based assays would be that SIRT1 deficient mice might display impaired adaptation to nutrient and energy stress. In line with this, muscle-specific SIRT1 KO mice failed to become more insulin sensitive upon CR [42]. CR increases SIRT1 deacetylase activity in skeletal muscle, in parallel with enhanced insulin-stimulated phosphoinositide 3-kinase (PI3K) signaling and glucose uptake [42]. These adaptations were completely abrogated in mice lacking SIRT1 deacetylase activity in muscle [42]. Mechanistically, this could be explained by various reasons. First, SIRT1 was found to be required for the deacetylation and inactivation of the transcription factor Stat3 during CR, which resulted in decreased gene and protein expression of the p55α/p50α subunits of PI3K, thereby promoting more efficient PI3K signaling during insulin stimulation [42]. Alternatively, SIRT1 has also been demonstrated to be a repressor of the protein tyrosine phosphatase 1B (PTP1b), a major tyrosine phosphatase for the insulin receptor and the insulin receptor substrate proteins, IRS1 and IRS2 [57]. Therefore, it is likely that SIRT1 deficient muscles also display higher PTP1b activity, which would also antagonize the augmentation of insulin signaling in response to CR. These results clearly support the notion that SIRT1 is key for metabolic adaptations triggered by nutrient deprivation in skeletal muscle (Figure 2). The effects of CR on insulin sensitivity are not, however, enhanced by muscle-specific overexpression of SIRT1 [40]. This might indicate that SIRT1 is required for the enhancement of insulin sensitivity induced by CR, but that the endogenous levels of SIRT1 are sufficient to maximize these effects.

Given the role of skeletal muscle SIRT1 in CR-induced adaptations, it was surprising to see that SIRT1 deficiency in skeletal muscle did not impair exercise training-induced metabolic adaptations [41,52]. However, exercise is a complex stimulus, affecting multiple pathways with likely redundant functions. Strikingly, PGC-1α was normally deacetylated in response to exercise, despite the lack of SIRT1 [52]. To solve this paradox, it was proposed that muscle contraction decreases the interaction of PGC-1α with the acetyltransferase enzyme, GCN5 [52]. This way, PGC-1α deacetylation upon exercise would not be a consequence of enhanced deacetylation, but of decreased acetylation rates. Interestingly, it was recently found that resveratrol had synergistic effects with exercise on muscle mitochondrial biogenesis [41]. While the effect of exercise on mitochondrial biogenesis was independent of SIRT1, the synergy with resveratrol was lost on SIRT1 muscle-specific KO mice [41].

Altogether, these results indicate that the absence of SIRT1 in skeletal muscle does not lead to any major defect in the basal state. While skeletal muscle SIRT1 is required for CR-induced adaptations at the level of insulin sensitivity, it seems dispensable for the benefits of other interventions such as exercise training.

## 5 SIRT1 roles in adipose tissues

### 5.1 White adipose tissue

White adipose tissue (WAT) is an important organ for the regulation of metabolic homeostasis, as it is the major fat depot in mammalian organisms. Fat storages are dynamically regulated in WAT. Lipolytic or lipogenic processes can be activated in response to nutrients and hormones [58]. Importantly, WAT also has critical actions as an endocrine tissue, by secreting hormones and cytokines, such as leptin, adiponectin or tumor necrosis factor α (TNFα), that affect insulin sensitivity, inflammation and, therefore, have major consequences on metabolic homeostasis [58].

One of the critical regulators of fat storage in WAT is the nuclear receptor PPARγ, whose activity promotes adipocyte differentiation and lipid anabolism [58]. A possible role of SIRT1 in WAT homeostasis was evidenced when it was found that SIRT1 could act as a PPARγ repressor [59]. During fasting, SIRT1 associated with PPARγ and promoted the binding of the nuclear receptor corepressor 1 (NCoR1) [59]. This favored fat mobilization instead of storage. A complementary study demonstrated that SIRT1 could repress PPARγ transcriptional activity on target lipogenic genes directly through deacetylation [60]. Ablation of SIRT1 in adipose tissue promotes body weight gain, mostly due to an increase in fat mass. The size of individual adipocytes was larger than in control mice, even on chow diet [61]. Altogether, this renders the adipocyte-specific SIRT1 KO mice prone to develop insulin resistance. Importantly, it has been described that obesity results in decreased SIRT1 levels, both in rodent and human adipose tissue [61–63]. The reason for this decrease might rely on the fact that obesity triggers the cleavage of SIRT1 through a caspase-1 dependent mechanism [61]. This cleavage renders SIRT1 prone to degradation, thereby decreasing SIRT1 activity.

**Figure 3:**
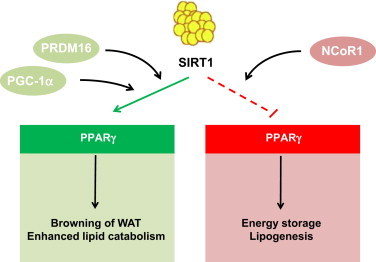
Schematic representation of the actions of SIRT1 in white adipose tissue, based on genetically engineered mouse models. SIRT1 activation can target PPARγ activity in white adipose tissue. On the one hand, SIRT1 docks the NCoR1 to PPARγ, repressing the expression of genes linked to lipid anabolism and storage. On the other, SIRT1 directly deacetylates PPARγ, which allows the recruitment of PRDM16 to drive “browning” of white fat. Similar actions might be mediated through the activation of PGC-1α by SIRT1.

Further confirming the key role of SIRT1 in adipose tissue homeostasis, a recent manuscript described how adipose-specific overexpression of human SIRT1 protects mice against age-related glucose intolerance [64]. This effect was concomitant with a prevention of fat accumulation in peripheral tissues and a higher ability of WAT to trigger lipolysis and fat oxidation [64]. Therefore, both the adipose tissue-specific overexpression and deletion models confirm that SIRT1 has a major role in preventing excessive fat accumulation and enhancing the ability of the tissue to respond to lipolytic stimuli (Figure 3). A caveat of both models resides in the use of the aP2-Cre line as a driver for recombination, as several reports indicate that this line leaks Cre recombinase expression into other tissues, including endothelial cells, brain, liver and skeletal muscle [65,66]. Hence, work with other Cre lines will be necessary to fully certify SIRT1 actions in adipose tissue.

Interestingly, the marked decrease in fat mass observed in adipose-tissue specific SIRT1 overexpressing mice was not observed in global SIRT1 overexpressing models. Indeed, two of the SIRT1 overexpressing models do not show any apparent difference in fat mass [4,5], while a third reported only a modest reduction [8]. The differences between the three models might stem from the different overexpression levels and sites. In the models produced by the Serrano and Accili labs [4,5], there is a mild whole body overexpression, while in the models reported by Bordone et al. the overexpression is present in adipose tissues and brain, but not in skeletal muscle or liver [8].

Adipose tissue inflammation is believed to be a hallmark of whole body insulin resistance. All animal models examined to date suggest a protective role of SIRT1 in adipose tissue inflammation [61,63]. SIRT1 represses the expression of genes implicated in inflammation in adipocytes [67]. Adipose-specific SIRT1 KO mice displayed increased macrophage recruitment to adipose tissue [63]. In line with these studies, overexpression of SIRT1 prevents against adipose tissue macrophage accumulation caused by HFD [63]. Importantly, it has also been shown in humans that SIRT1 mRNA levels are inversely related to adipose tissue macrophage infiltration in sub-cutaneous fat [63].

### 5.2 Brown and beige adipose tissue

Brown adipocytes are characterized by the expression of mitochondrial uncoupling protein 1 (UCP1), which allows dissipation of energy as heat for thermogenesis [58]. It has become apparent that brown adipocytes in different depots might derive from independent precursors. Hence, brown adipocytes located in the prototypical interscapular brown adipose tissue (BAT) seem to have a common precursor with skeletal muscle cells [68]. In contrast, brown adipocytes residing in WAT share a precursor with white adipocytes and have an intermediate phenotype between canonical brown and white adipocytes, hence their designation as brite or beige adipocytes [69]. In response to adrenergic stimulation or cold exposure, white adipocytes can also obtain brown adipocyte-like characteristics, turning into beige adipocytes [58,69–72]. The binding of PGC-1α to PPARγ promotes brown adipocyte-like features in white adipocytes though an upregulation of brown-adipocyte specific genes, such as UCP1, and a down-regulation of white-adipocyte specific genes [72]. Adipose tissue specific SIRT1 KO mice display both enhanced WAT and BAT mass, due to enhanced fat accumulation [61]. Given the ability of SIRT1 to increase PGC-1α activity and lipid oxidation, SIRT1 activation might prevent excessive accumulation of fat in adipocytes by boosting fat consumption and enhancing thermogenic function. Indeed, a recent study has demonstrated a role for SIRT1 in the “browning” of WAT. Overexpression of SIRT1 results in a down-regulation of WAT specific genes in WAT depots and up-regulates BAT characteristics, while SIRT1 deletion has the opposite effect [60]. This is mediated by SIRT1-dependent deacetylation of PPARγ which facilitates the recruitment of PRDM16, a transcriptional coregulator that drives the BAT adipogenic program [73]. Consequently, mice overexpressing SIRT1 have a more potent induction of a BAT-like phenotype of subcutaneous WAT upon cold exposure [60].

Overall, while we are just beginning to understand the influence of SIRT1 on adipose tissue homeostasis, it seems clear that SIRT1 exacerbates the induction of lipid mobilization and WAT-browning effect induced by cold exposure (Figure 3). Therefore, SIRT1 activation in the adipose tissue could protect against metabolic diseases by enhancing energy expenditure and favoring thermogenic function. A limitation for these studies has been the absence of a proper Cre-line to drive recombination specifically in brown adipocytes. Such a model has been recently reported [74] and might enhance our understanding of the role of SIRT1 in canonical brown fat.

## 6 SIRT1 and the endocrine pancreas

Pancreatic β-cells play a central role in the regulation of glucose homeostasis by secreting insulin in response to elevated glucose levels. Initial data from transgenic models proposed that SIRT1 positively controls glucose stimulated insulin secretion (GSIS). Overexpression of SIRT1 specifically in pancreatic β-cells is sufficient to improve glucose tolerance and insulin secretion in response to glucose or depolarization (KCl) [75]. In line with this model, GSIS is blunted in islets from SIRT1 KO mice or in β-cells where SIRT1 has been knocked down by siRNAs [76]. Both studies propose that SIRT1 improves GSIS though the downregulation of mitochondrial uncoupling protein 2 (UCP2). This would enhance ATP production in response to elevated glucose levels. Along this line of reasoning, β-cell specific overexpression of SIRT1 alone prevents HFD-induced glucose intolerance. The beneficial effects of SIRT1 on insulin secretion, however, are progressively diminished upon aging and completely lost at 18–24 months [77]. This was explained by a decrease of NAD^+^ availability in aged tissues, which could potentially limit the activity of SIRT1. Supporting this hypothesis, increasing NAD^+^ by nicotinamide mononucleotide (NMN) supplementation rescues the benefits of β-cell SIRT1 overexpression in aged mice [77]. In line with the beneficial effects of SIRT1 on pancreatic function, SIRT1 activation could also be a promising strategy to prevent the deleterious effects of lipotoxicity on insulin secretion and β-cell function [78]. The requirement of SIRT1 for proper adult pancreatic β-cell function was finally consolidated by a recent report using floxed SIRT1 mice crossed with the *Pdx1-CreER* deleter strain [79]. In line with previous findings, SIRT1 deficiency impaired insulin secretion by disrupting glucose sensing [79]. There were no apparent defects in insulin content or β-cell mass upon the deletion of SIRT1, but they failed to properly respond to fluctuations in glucose levels [79]. Importantly, the lack of response did not stem from enhanced *Ucp2* expression or mitochondrial uncoupling, but from impaired overall mitochondrial function, which decreased the ability of mitochondria to synthesize ATP in response to a glucose load [79].

Pancreatic β-cell function can be also influenced by controlling β-cell mass, determined by the balance between apoptotic, proliferative and neogenic processes. Pancreatic β-cell mass and β-cell area are unchanged in β-cell specific SIRT1 overexpressing mice as well as in heterozygous and homozygous SIRT1 KO mice [75,76]. The lack of effect of SIRT1 on pancreatic β-cell mass, however, has been recently challenged by a few observations. Firstly, deletion of the poly(ADP-ribose) polymerase (PARP)-2 gene leads to a constitutive increase in SIRT1 expression in many tissues, including pancreas [80]. *PARP-2*^*−/−*^ mice display marked glucose intolerance despite being more insulin sensitive [80]. Explaining this, GSIS is dramatically impaired in *PARP-2* deficient mice. Upon close examination *β*-cell mass was significantly reduced in *PARP-2*^*−/−*^ mice, and failed to proliferate upon high-fat feeding, augmenting HFD-induced glucose intolerance [80]. Mechanistically, it was proposed that higher SIRT1 activity led to a constitutive deacetylation and activation of FoxO1. In turn, FoxO1 is a negative regulator of β-cell expansion through repression of the pancreatic and duodenal homobox 1 (PDX1) transcription factor, which is a critical regulator of β-cell proliferation and differentiation [81]. Accordingly, the expression of PDX1 and its target genes was dramatically reduced in *PARP-2* deficient mice [80]. Likewise, GLP-1 positively influences β-cell proliferation by disrupting the association between FoxO1 and SIRT1 [82]. This promotes FoxO1 hyperacetylation and nuclear exclusion, thereby relieving the repression of pancreatic β-cell proliferation. This constitutes another example where SIRT1 is also regarded as a negative regulator of β-cell mass and where SIRT1 inhibition might actually be beneficial to enhance β-cell function.

These seemingly opposite effects might have an explanation. While chronic activation of SIRT1 can be deleterious for β-cell expansion, SIRT1 might be required for the appropriate protection and adaptation of β-cells to oxidative stress and inflammation. Indeed, SIRT1 has been shown to be protective against cytokine induced β-cell toxicity [83]. This way, SIRT1 might have both protective and detrimental roles on β-cell function, depending on the timing and flexibility of its activation. Therefore, therapeutic approaches aimed to increase SIRT1 activity in β-cells should take into account this delicate balance.

## 7 SIRT1 and food intake behavior

Hypothalamic neurons are able to detect changes in circulating hormones and nutrients and respond to these changes by secreting several hunger/satiety hormones such as the *α*-melanocyte-stimulating hormone (*α*-MSH) or the agouti-related protein (AgRP). The pro-opiomelanocortin (POMC) expressing neurons and AgRP expressing neurons in the hypothalamus constitute central nodes in the regulation of feeding behavior and energy expenditure. POMC neurons promote satiety through the release of *α*-MSH, which is a ligand for melanocortin-4 receptor (MC4R) bearing neurons. Conversely, AgRP is an antagonist for the MC4R and promotes food intake in response to fasting or CR [84].

SIRT1 is highly expressed in the arcuate nucleus (ARC), where we find AgRP and POMC neurons, and in the ventromedial nuclei (VMN), where we find the MC4R neurons [85,86]. Selective deletion of SIRT1 in AgRP neurons is sufficient to decrease food intake due to impaired MC4R antagonism [87]. This result suggests that SIRT1 activity in this neuronal population might be required to increase food intake in situations of nutrient deprivation. Strikingly, no effects on food intake where observed when SIRT1 gene was deleted in POMC neurons [88]. However, mice lacking SIRT1 in POMC neurons were prone to obesity upon high-fat feeding. This effect, however, does not seem to stem from the control of food intake behavior but, rather, by indirectly decreasing the metabolic rate of peripheral tissues [88].

Whole brain overexpression of SIRT1 enhances ambulatory activity in response to CR [86]. Oppositely, whole brain deletion of SIRT1 prevents the increase in activity generally triggered by CR [89]. This observation suggests SIRT1 could play a role in the control of pituitary hormones and consequently have metabolic effects in response to different dietary challenges. Again, this highlights how SIRT1 might be activated upon nutrient scarcity and promotes adaptations aimed to enhance food foraging behavior. However, one should be cautious when extrapolating these results to the human scenario, given the very different feeding-related behaviors in both species. Furthermore, it has been recently proposed that whole brain overexpression might actually extend lifespan and delay aging, even though this might depend on a delicate balance between the levels of SIRT1 overexpression in different brain regions [90]. This balance between SIRT1 activities in different brain regions, at the same time, might explain why the brain-specific SIRT1 overexpressing mice, but not the global SIRT1 overexpressing mice, display lifespan extension. Furthermore, the regulation of endogenous SIRT1 activity in the hypothalamus remains unclear. While SIRT1 expression and activity might be modulated by food intake, a couple of studies have led to apparently opposite conclusions. On the one hand, it was reported that fasting increased SIRT1 activity in the hypothalamus, inducing the deacetylation of FoxO. This, in turn, repressed POMC neurons and enhanced AgRP expression, promoting food intake [91]. Another study, however, demonstrated that hypothalamic SIRT1 protein levels decrease during fasting [92]. In this case, the authors argue that SIRT1 inhibits FoxO1-dependent expression of AgRP and consequently leads to the cessation of feeding [92]. While these two studies illustrate the relevant nature of the SIRT1-FoxO1 axis for the regulation of food intake, the intricacy of the system and apparently opposite observations need to be resolved. Recent reports have indicated that SIRT1 might also influence circadian rhythm regulation and as a result, indirectly impact on metabolic health and the aging process [93].

## 8 SIRT1 in physiology: comparing and contrasting different approaches

Our knowledge of the physiological roles of SIRT1 arises not only from direct genetic manipulations of the SIRT1 gene, but also through the modulation of SIRT1 activity by multiple means.

### 8.1 Pharmacological approaches

A number of compounds have been used as SIRT1 activating compounds (STACs), even though their specificity has been long debated [1,94]. A recent publication has suggested that STACs can directly influence the activity of SIRT1 through a specific residue, Glu^230^ for human SIRT1 [95]. However, this mechanism for direct activation of SIRT1would only affect a subset of substrates with specific structural requirements [95] and would not explain the effects of STACs on lower eukaryotes, where the human Glu^230^ residue is not conserved. Therefore, further *in vivo* confirmation of these observations will be required to prove beyond doubt that STACs action can be attributed solely to SIRT1. Among all STACs, resveratrol has probably been the one receiving most attention. Resveratrol, a natural polyphenol, was identified a decade ago as a direct activator of SIRT1 [96]. A more recent screen for other possible small molecular SIRT1 agonists led to the identification of a second batch of compounds, among which the best characterized is SRT1720 [97]. Mice administered resveratrol or SRT1720 show a number of features in common with SIRT1 transgenic mice. Most notably, they are protected against HFD-induced glucose intolerance and display enhanced mitochondrial function [6,7,98,99]. Both compounds significantly prevented the decrease in lifespan prompted by HFD [6,7]. However, some indications cast a shadow of doubt on the specificity of these compounds on SIRT1. SRT1720, while being a dramatically more potent SIRT1 activator in the *in vitro* assays, required similar doses to those of resveratrol to achieve health benefits. Also, the effects of STACs on energy metabolism were more marked than those observed in SIRT1 transgenic models. For example, both compounds largely prevented HFD-induced obesity [98,99], an effect never observed in SIRT1 transgenic mice [4,5]. Similarly, both compounds had major effects on skeletal muscle physiology, allowing mice to run longer on a treadmill test when used at relatively high doses [98,99], while SIRT1 overexpressing mice do not run longer than WT littermates (Boutant M, Canto C, unpublished observations). A possible explanation for these discrepancies relies on the ability of these compounds to activate other pathways [1,6,99,100]. Nonetheless, resveratrol has been reported to directly bind a number of transcriptional regulators (see [101] for review) and components of the mitochondrial respiratory chain [102], leading to decreased ATP production and, consequently, higher AMPK activity [103]. In fact, many studies suggest that resveratrol requires AMPK to activate SIRT1 and achieve health benefits in *in vivo* settings [46,104], at least in the doses most commonly used in animal studies [9]. Indeed, AMPK activation through other pharmacological means has provided very similar outputs to those described for STACs, such as enhanced endurance capacity [105] and amelioration of glycaemic profiles upon high-fat feeding or in genetic models of obesity [106].

Given the fast generation of a constellation of genetically-engineered mouse models, pharmacological inhibition of SIRT1 has not been studied systematically in rodents. The most widely used inhibitor for SIRT1 is EX-527. While metabolic analyses after chronic EX-527 treatment have not been reported, some studies have used EX-527 in a more acute or local fashion. For example, intracerebroventricular injection of EX-527 leads to reduced food intake in rodents, mirroring the results obtained when injecting SIRT1 siRNAs or deleting SIRT1 in AgRP neurons [87,91].

### 8.2 The modulation of SIRT1 interacting proteins

SIRT1 activity can also be modulated by affecting its interaction with regulatory proteins. Different groups identified the nuclear protein DBC1 as a protein that forms a stable complex with SIRT1 *in vivo* and *in vitro* [107–109]. DBC1 binds to the catalytic domain of SIRT1, perturbing SIRT1 activity [107,108]. This interaction is dynamically regulated. Under normal circumstances, around 50% of total SIRT1 in liver is associated with DBC1, but this interaction is essentially lost upon starvation, when SIRT1 activity is higher [109]. In contrast, the interaction was more prominent on HFD, when SIRT1 activity is decreased [109]. It has been recently reported that this interaction can be modulated by phosphorylation events. For example, the interaction between DBC1 and SIRT1 increases following DNA damage and oxidative stress [110]. This is due to the phosphorylation of DBC1 at Thr^454^ by the ATM (ataxia telangiectasia-mutated) and ATR (ataxia telangiectasia and Rad3-related) kinases, which create a second binding site for SIRT1 [110,111]. In contrast, it has been found that activation of the AMPK pathway leads to the dissociation of DBC1–SIRT1 complexes [47], even though the underlying mechanism is not fully understood. Mice lacking DBC1 display a 2- to 4-fold increase in SIRT1 activity in a wide range of tissues [109]. Consequently DBC1 KO mice share many features with SIRT1 transgenic mice, such as protection against HFD-induced hepatic steatosis and inflammation [109]. However, DBC1 deficient mice still developed diabetes under high-fat feeding [109], indicating that DBC1 might be affecting additional cellular functions other than those related to SIRT1.

Conversely, the active regulator of SIRT1 (AROS) was identified as a positive regulator of SIRT1 activity [112]. The interaction of AROS with SIRT1, presumably with its catalytic domain, enhances SIRT1 activity by 2-fold [112]. The ability of AROS to directly modulate SIRT1 activity, however, has been recently challenged [113], and no mouse models have been generated in order to test the physiological impact of AROS on SIRT1.

### 8.3 Enhancing NAD^+^ availability

Most, if not all, experimental strategies designed to alter intracellular NAD^+^ levels have consistently been shown to influence SIRT1 activity in cultured cell models [1]. Consequently, a number of strategies have been devised to test whether increases in NAD^+^ availability might translate into SIRT1 activation in animal models. One strategy relied on slowing NAD^+^ breakdown by the deletion of different NAD^+^ consumers. PARP-1 is considered to be a major NAD^+^ consumer in the cell, and its activity can deplete intracellular NAD^+^ by 70% [114]. The deletion of PARP-1 is sufficient to increase basal NAD^+^ availability and SIRT1 activity [115]. PARP-1 deficient mice also show a number of phenotypes resembling SIRT1 transgenesis and pharmacological activation, most notably a remarkable protection against HFD-induced insulin resistance [115]. A similar case could be made for CD38, another avid cellular NAD^+^ consumer [116]. Mice defective for CD38 have increased SIRT1 activity in most tissues, likely due an almost 20-fold increase in NAD^+^ availability [117]. This confers protection against many of the metabolic complications induced by HFD [117]. However, both PARP-1 and CD38 total KO models display a more pronounced protection against HFD comorbidities than that observed in SIRT1 transgenic mice. For example, both PARP-1 and CD38 deletion prevent body weight gain upon high-fat feeding, while SIRT1 overexpression does not. This could be due to two reasons: first, that these alternative NAD^+^ consumers affect many processes other than SIRT1 activity and, second, that SIRT1 transgenesis might not reach comparable SIRT1 activity levels as in the PARP-1 or CD38 models, due to NAD^+^ availability limitations.

A second strategy to enhance NAD^+^ availability consists in enhancing NAD^+^ synthesis by providing NAD^+^ precursors or manipulating the expression of NAD^+^ biosynthetic enzymes. Intraperitoneal injections of NMN are sufficient to raise NAD^+^ levels in key metabolic tissues and robustly increase SIRT1 activity [118]. After seven days of treatment, a strong amelioration in glycaemic profiles could be observed in HFD- and age-related models of glucose intolerance [118]. Parallel experiments demonstrated that NMN prevented fructose rich diet-induced islet dysfunction, likely through SIRT1 activation [119]. Similarly, dietary supplementation with nicotinamide riboside (NR), a natural NAD^+^ precursor that can be found in food and beverages, also led to a robust elevation in NAD^+^ levels and enhanced SIRT1 activity in mouse tissues [120]. This was coupled to a marked enhancement of insulin sensitivity, in both chow and high-fat fed animals, as well as increased oxidative capacity and global energy expenditure [120]. Overall, these strategies illustrate how the administration of NAD^+^ precursors constitutes an excellent tool to enhance SIRT1 activity *in vivo* and recapitulate the health benefits of SIRT1 overexpression, *e.g.* a strong protection against insulin resistance and increased oxidative capacity. However, it must be stressed that SIRT1 might be just one of the many mechanism by which NAD^+^ precursors prompt health benefits in rodents. Experiments using SIRT1 deficient models will be required to evaluate the role of SIRT1 in the actions of NMN or NR.

The overexpression of NAD^+^ biosynthetic enzymes, such as Nampt or the NMN adenylyltransferase 1 (NMNAT1), provides an alternative way to boost NAD^+^ availability, which generally leads to enhanced SIRT1 activity [121–124]. In fact, it has been reported that SIRT1 might directly interact with NMNAT1 [123]. Such a complex could potentially channel NAD^+^ production to fuel SIRT1 enzymatic catalysis, creating a microdomain for the regulation of SIRT1 activity. Supporting this notion, the Slow Wallerian Degeneration (Wld^S^) spontaneous mutant mice, overexpressing a chimeric protein that contains the full length NMNAT1 protein, display enhanced NAD^+^ availability and are protected against streptozotocin- and dietary-induced glucose intolerance in a SIRT1 dependent manner [124].

Interestingly, a recent publication suggests that it might be the metabolites produced by SIRT1-mediated NAD^+^ breakdown what drive some of the most notable physiological effects, such as lifespan extension in low eukaryotes [125]. The authors propose that the generation of 1-methylnicotinamide from nicotinamide, one of the end-products of the sirtuin reaction, leads to a mitohormetic signal that culminates in worm lifespan extension [125]. Indeed, the blockage of 1-methylnicotinamide production abolished the effects of SIR2.1 overexpression on lifespan [125]. Conversely, supplementation with 1-methylnicotinamide was enough to enhance *C. elegans* lifespan [125]. While mammalian translation of these findings will be necessary, these results might help calling our attention on the largely underappreciated role of the metabolites derived from SIRT1 catalytic activity as second messengers.

## 9 Conclusions and future perspectives

In this review we have provided an update on the physiological actions of SIRT1 based on the lessons learned from transgenic mouse models (Tables 1 and 2). Most data support the notion that SIRT1 contributes to an efficient adaptation of cellular and organismal metabolism to nutritional and energy status. The activation of SIRT1 can be intimately linked to cellular metabolism by the rate-limitation imposed by NAD^+^ bioavailability. Furthermore, recent evidence indicates that SIRT1 might not just act as a deacetylase enzyme, but also as an efficient deacylase enzyme for short, mid and long-chain fatty acids [126]. This opens a whole new universe for possible SIRT1 functions and emphasizes the intimate link between SIRT1 and fatty acid metabolism. In general, SIRT1 activation is linked to a more efficient use of lipid energy sources and respiratory metabolism. This way, SIRT1 deletion in different tissues often leads to abnormal fat accumulation due to deficient lipid catabolism.

The physiological regulation of SIRT1 activity is complex, and impacts on a wide network of substrates whose regulation is not solely controlled by SIRT1 itself. This can explain why initial findings on cultured cell models of SIRT1 overexpression or downregulation have sometimes not clearly mirrored mouse physiology, where changes in SIRT1 activity might be more subtle or subject to temporal control. Another caveat of our understanding of SIRT1 comes from the knowledge inferred through the use of resveratrol or other STACs, whose specificity of action is not fully clear. Overall, the collection of mouse models generated to date, have largely clarified the true scope, role and limitations of SIRT1 actions. First, they established SIRT1 as a key gene for correct early organismal development. Tissue or temporally-controlled transgenic models all converge on the key role of SIRT1 for metabolic efficiency. While the claims of SIRT1 as a “longevity” gene are debatable, the transgenic models indicate that SIRT1 can certainly impact on health and age-related physiological decline in a pleiotropic manner. Interestingly, tissue-specific deletions or overexpression have highlighted how the contribution of SIRT1 to metabolic homeostasis in different tissues is substantially different. A striking case is skeletal muscle, where deletion or overexpression of SIRT1 does not seem to have any major effect on global metabolic homeostasis in the basal state. In contrast, most models for liver- or adipose tissue-specific SIRT1 deletion all converge in altered lipid anabolism or catabolism. It will be important to understand why some tissues are more sensitive to changes in SIRT1 expression than others. Potential reasons for this might rely on different linearity between SIRT1 expression and activity levels, as well as on the tissue-specific cohabitation of SIRT1 with other regulatory proteins.

On the therapeutic side, the administration of NAD^+^ precursors is rising as a promising strategy to activate SIRT1 and improve glucose homeostasis in insulin resistant profiles. Interestingly, a recent report indicates that NAD^+^ precursors can enhance healthspan in worms [127]. This perfectly mirrors the observations in mammals and testifies for an evolutionarily conserved ability of these compounds to provide health benefits.

Thus, SIRT1 holds its place as an extremely attractive target to improve oxidative metabolism and mitochondrial function which are generally impaired in insulin resistant and aged populations. However, fine-tuned SIRT1 activity might be critical to fully exploit these metabolic advantages. This is epitomized by the pancreatic regulation of SIRT1 activity, where constitutive activation of SIRT1 has been reported to be detrimental for global glucose tolerance. Likewise, while SIRT1 has been shown to be protective against oxidative stress and ischemia/reperfusion in cardiac muscle [128,129], enhanced SIRT1 activity in the heart can lead to cardiac failure by promoting dilated cardiomyopathy [129–131]. Indeed, too much of a good thing might not always be desirable. Critical balances between metabolic benefits and side-effects might need to be struck regarding pharmacological approaches aimed to increase SIRT1 activity.

## Conflict of interest

M.B. and C.C. are employees of the Nestlé Institute of Health Sciences S.A.

## Figures and Tables

**Table 1:** Prominent energy metabolism phenotypes observed in SIRT1 gain-of-function models through genetic mechanisms.

**Targeted tissue**	**Genetic strategy**	**Prominent phenotypes observed**	**References**
Whole body	Overexpression (moderate)	Protection against dietary and age-related metabolic damage.	Pfluger et al. [4]
			Banks et al. [5]
		Similar lifespan as WT mice	Herranz et al. [3]
	Overexpression (moderate)	Calorie-restriction like behavior	Bordone et al. [8]
	Overexpression (moderate)	Higher susceptibility to atherosclerotic lesions when fed a atherogenic diet	Quiang et al. [60]
	Overexpression (high)	Higher muscle mitochondrial content	Price et al. [9]
Liver	Overexpression (adenoviral delivery)	Positive regulation of hepatic glucose production and inhibition of lipid anabolism	Rodgers et al. [21]
	Overexpression (adenoviral delivery)	Attenuation of hepatic glucose production and insulin resistance in ob/ob mice	Wang et al. [20]
Muscle	Overexpression	Similar aspect, insulin sensitivity and adaptation to calorie restriction as in wild-type mice	White et al. [40]
	MCK-Cre		
Adipose tissue	Overexpression	Prevention against age-induced deterioration of insulin sensitivity and ectopic lipid distribution. Reduction of whole body fat mass and enhanced locomotor activity	Xu et al. [64]
	Ap2-Cre		
Pancreas	Overexpression	Enhanced glucose-induced insulin secretion	Moynihan et al. [75]
	SIRT1 insertion under the human insulin promoter		
Brain	Whole brain overexpression	Enhanced foraging behavior upon calorie restriction	Satoh et al. [86,90]
	SIRT1 insertion under the mouse PrP promoter	Lifespan extension	

**Table 2:** Prominent energy metabolism phenotypes observed in SIRT1 loss-of-function models through genetic mechanisms.

Targeted tissue	Genetic strategy	Prominent phenotypes observed	References
Whole body	Knock-out	High embryonic lethality	McBurney et al. [12]
		Numerous developmental defects	
	Knock-out	Numerous developmental defects	Cheng et al. [13]
		Infrequent postnatal survival	
	Knock-out (outbred stocks)	Metabolic inefficiency and defective adaptation to nutrient stress	Boily et al. [14]
	Knock-out (Adulthood deletion)	Defective mitochondrial function	Price et al. [9]
	Hemizygosis	Hepatic steatosis	Purushotham et al. [15]
			Xu et al. [64]
Liver	Deletion	Protection from physiological decline when fed a high-fat diet	Chen et al. [18]
	Alb-Cre; SIRT1 fl/fl (exon 4)		
	Deletion	Higher susceptibility for the development of hepatosteatosis	Purushotham et al. [9]
	Alb-Cre; SIRT1 fl/fl (exon 4)		
	Deletion	Hepatic steatosis even on chow diet and chronic hyperglycemia	Wang et al. [17,20]
	Alb-Cre; SIRT1 fl/fl (exon 5–6)		
Muscle	Deletion	Normal adaptation to exercise, but not to calorie restriction	Schenk et al. [42]
	MCK-Cre; SIRT1 fl/fl (exon 4)		Philp et al. [52]
	Deletion	Defective mitochondrial function	Menzies et al. [41]
	MLC1f-Cre; SIRT1 fl/fl (exon 4)	No synergism between resveratrol and exercise on mitochondrial biogenesis	
Adipose tissue	Deletion	Increased inflammation of white adipose tissue, increased adiposity and higher susceptibility to obesity and insulin resistance	Gillum et al. [63]
	FABP4-Cre; SIRT1 fl/fl (exon 4)		Chalkiadaki et al. [61]
Pancreas	Adulthood deletion	Disrupted glucose-stimulated insulin secretion	Luu et al. [79]
	Pdx1-ERCre; SIRT1 fl/fl (exon 4)		
Brain	Whole brain deletion	Altered behavioral response to caloric restriction	Cohen et al. [89]
	Nestin-Cre; SIRT1 fl/fl (exon 4)	Defective control of pituitary hormones	
		Increased glucose intolerance with aging	
	Deletion in AgRP neurons	Decreased food intake and body weight	Dietrich et al. [87]
	Agrp-Cre; SIRT1 fl/fl (exon 4)		
	Deletion in POMC neurons	Hypersensitivity to HFD-induced obesity	Ramadori et al. [88]
	POMC-Cre; SIRT1 fl/fl (exon 4)		

## References

[1] Canto C., Auwerx J. (2012). Targeting sirtuin 1 to improve metabolism: all you need is NAD(+)?. Pharmacological Reviews.

[2] Lombard D.B., Pletcher S.D., Canto C., Auwerx J. (2011). Ageing: longevity hits a roadblock. Nature.

[3] Herranz D., Munoz-Martin M., Canamero M., Mulero F., Martinez-Pastor B., Fernandez-Capetillo O. (2010). Sirt1 improves healthy ageing and protects from metabolic syndrome-associated cancer. Nature Communications.

[4] Pfluger P.T., Herranz D., Velasco-Miguel S., Serrano M., Tschop M.H. (2008). Sirt1 protects against high-fat diet-induced metabolic damage. Proceedings of the National Academy of Sciences of the United States of America.

[5] Banks A.S., Kon N., Knight C., Matsumoto M., Gutierrez-Juarez R., Rossetti L. (2008). SirT1 gain of function increases energy efficiency and prevents diabetes in mice. Cell Metabolism.

[6] Baur J.A., Pearson K.J., Price N.L., Jamieson H.A., Lerin C., Kalra A. (2006). Resveratrol improves health and survival of mice on a high-calorie diet. Nature.

[7] Minor R.K., Baur J.A., Gomes A.P., Ward T.M., Csiszar A., Mercken E.M. (2011). SRT1720 improves survival and healthspan of obese mice. Scientific Reports.

[8] Bordone L., Cohen D., Robinson A., Motta M.C., van Veen E., Czopik A. (2007). SIRT1 transgenic mice show phenotypes resembling calorie restriction. Aging Cell.

[9] Price N.L., Gomes A.P., Ling A.J., Duarte F.V., Martin-Montalvo A., North B.J. (2012). SIRT1 is required for AMPK activation and the beneficial effects of resveratrol on mitochondrial function. Cell Metabolism.

[10] Gerhart-Hines Z., Dominy J.E., Blattler S.M., Jedrychowski M.P., Banks A.S., Lim J.H. (2011). The cAMP/PKA pathway rapidly activates SIRT1 to promote fatty acid oxidation independently of changes in NAD(+). Molecular Cell.

[11] Braidy N., Guillemin G.J., Mansour H., Chan-Ling T., Poljak A., Grant R. (2011). Age related changes in NAD+ metabolism oxidative stress and Sirt1 activity in wistar rats. PLoS One.

[12] McBurney M.W., Yang X., Jardine K., Hixon M., Boekelheide K., Webb J.R. (2003). The mammalian SIR2alpha protein has a role in embryogenesis and gametogenesis. Molecular and Cellular Biology.

[13] Cheng H.L., Mostoslavsky R., Saito S., Manis J.P., Gu Y., Patel P. (2003). Developmental defects and p53 hyperacetylation in Sir2 homolog (SIRT1)-deficient mice. Proceedings of the National Academy of Sciences of the United States of America.

[14] Boily G., Seifert E.L., Bevilacqua L., He X.H., Sabourin G., Estey C. (2008). SirT1 regulates energy metabolism and response to caloric restriction in mice. PLoS One.

[15] Purushotham A., Xu Q., Li X. (2012). Systemic SIRT1 insufficiency results in disruption of energy homeostasis and steroid hormone metabolism upon high-fat-diet feeding. FASEB Journal.

[16] Xu F., Gao Z., Zhang J., Rivera C.A., Yin J., Weng J. (2010). Lack of SIRT1 (Mammalian Sirtuin 1) activity leads to liver steatosis in the SIRT1+/− mice: a role of lipid mobilization and inflammation. Endocrinology.

[17] Wang R.H., Kim H.S., Xiao C., Xu X., Gavrilova O., Deng C.X. (2011). Hepatic Sirt1 deficiency in mice impairs mTorc2/Akt signaling and results in hyperglycemia, oxidative damage, and insulin resistance. Journal of Clinical Investigation.

[18] Chen D., Bruno J., Easlon E., Lin S.J., Cheng H.L., Alt F.W. (2008). Tissue-specific regulation of SIRT1 by calorie restriction. Genes Dev..

[19] Purushotham A., Schug T.T., Xu Q., Surapureddi S., Guo X., Li X. (2009). Hepatocyte-specific deletion of SIRT1 alters fatty acid metabolism and results in hepatic steatosis and inflammation. Cell Metabolism.

[20] Wang R.H., Li C., Deng C.X. (2010). Liver steatosis and increased ChREBP expression in mice carrying a liver specific SIRT1 null mutation under a normal feeding condition. International Journal of Biological Sciences.

[21] Rodgers J.T., Puigserver P. (2007). Fasting-dependent glucose and lipid metabolic response through hepatic sirtuin 1. Proceedings of the National Academy of Sciences of the United States of America.

[22] Li Y., Xu S., Giles A., Nakamura K., Lee J.W., Hou X. (2011). Hepatic overexpression of SIRT1 in mice attenuates endoplasmic reticulum stress and insulin resistance in the liver. FASEB Journal.

[23] Liu Y., Dentin R., Chen D., Hedrick S., Ravnskjaer K., Schenk S. (2008). A fasting inducible switch modulates gluconeogenesis via activator/coactivator exchange. Nature.

[24] Rodgers J.T., Lerin C., Haas W., Gygi S.P., Spiegelman B.M., Puigserver P. (2005). Nutrient control of glucose homeostasis through a complex of PGC-1alpha and SIRT1. Nature.

[25] Herzig S., Long F., Jhala U.S., Hedrick S., Quinn R., Bauer A. (2001). CREB regulates hepatic gluconeogenesis through the coactivator PGC-1. Nature.

[26] Yoon J.C., Puigserver P., Chen G., Donovan J., Wu Z., Rhee J. (2001). Control of hepatic gluconeogenesis through the transcriptional coactivator PGC-1. Nature.

[27] Yubero P., Hondares E., Carmona M.C., Rossell M., Gonzalez F.J., Iglesias R. (2004). The developmental regulation of peroxisome proliferator-activated receptor-gamma coactivator-1alpha expression in the liver is partially dissociated from the control of gluconeogenesis and lipid catabolism. Endocrinology.

[28] Canto C., Auwerx J. (2010). AMP-activated protein kinase and its downstream transcriptional pathways. Cellular and Molecular Life Sciences.

[29] Puigserver P., Rhee J., Donovan J., Walkey C.J., Yoon J.C., Oriente F. (2003). Insulin-regulated hepatic gluconeogenesis through FOXO1-PGC-1alpha interaction. Nature.

[30] Rhee J., Inoue Y., Yoon J.C., Puigserver P., Fan M., Gonzalez F.J. (2003). Regulation of hepatic fasting response by PPARgamma coactivator-1alpha (PGC-1): requirement for hepatocyte nuclear factor 4alpha in gluconeogenesis. Proceedings of the National Academy of Sciences of the United States of America.

[31] Walker A.K., Yang F., Jiang K., Ji J.Y., Watts J.L., Purushotham A. (2010). Conserved role of SIRT1 orthologs in fasting-dependent inhibition of the lipid/cholesterol regulator SREBP. Genes Development.

[32] Ponugoti B., Kim D.H., Xiao Z., Smith Z., Miao J., Zang M. (2010). SIRT1 deacetylates and inhibits SREBP-1C activity in regulation of hepatic lipid metabolism. Journal of Biological Chemistry.

[33] Li X., Zhang S., Blander G., Tse J.G., Krieger M., Guarente L. (2007). SIRT1 deacetylates and positively regulates the nuclear receptor LXR. Molecular Cell.

[34] Kemper J.K., Xiao Z., Ponugoti B., Miao J., Fang S., Kanamaluru D. (2009). FXR acetylation is normally dynamically regulated by p300 and SIRT1 but constitutively elevated in metabolic disease states. Cell Metabolism.

[35] Purushotham A., Xu Q., Lu J., Foley J.F., Yan X., Kim D.H. (2012). Hepatic deletion of SIRT1 decreases hepatocyte nuclear factor 1alpha/farnesoid X receptor signaling and induces formation of cholesterol gallstones in mice. Molecular and Cellular Biology.

[36] Kalaany N.Y., Mangelsdorf D.J. (2006). LXRS and FXR: the yin and yang of cholesterol and fat metabolism. Annual Review of Physiology.

[37] Qiang L., Lin H.V., Kim-Muller J.Y., Welch C.L., Gu W., Accili D. (2011). Proatherogenic abnormalities of lipid metabolism in SirT1 transgenic mice are mediated through Creb deacetylation. Cell Metabolism.

[38] Fulco M., Schiltz R.L., Iezzi S., King M.T., Zhao P., Kashiwaya Y. (2003). Sir2 regulates skeletal muscle differentiation as a potential sensor of the redox state. Molecular Cell.

[39] Fulco M., Cen Y., Zhao P., Hoffman E.P., McBurney M.W., Sauve A.A. (2008). Glucose restriction inhibits skeletal myoblast differentiation by activating SIRT1 through AMPK-mediated regulation of Nampt. Developmental Cell.

[40] White A.T., McCurdy C.E., Philp A., Hamilton D.L., Johnson C.D., Schenk S. (2013). Skeletal muscle-specific overexpression of SIRT1 does not enhance whole-body energy expenditure or insulin sensitivity in young mice. Diabetologia.

[41] Menzies K.J., Singh K., Saleem A., Hood D.A. (2013). Sirtuin 1-mediated effects of exercise and resveratrol on mitochondrial biogenesis. Journal of Biological Chemistry.

[42] Schenk S., McCurdy C.E., Philp A., Chen M.Z., Holliday M.J., Bandyopadhyay G.K. (2011). Sirt1 enhances skeletal muscle insulin sensitivity in mice during caloric restriction. Journal of Clinical Investigation.

[43] Gurd B.J. (2011). Deacetylation of PGC-1alpha by SIRT1: importance for skeletal muscle function and exercise-induced mitochondrial biogenesis. Applied Physiology, Nutrition, and Metabolism.

[44] Gerhart-Hines Z., Rodgers J.T., Bare O., Lerin C., Kim S.H., Mostoslavsky R. (2007). Metabolic control of muscle mitochondrial function and fatty acid oxidation through SIRT1/PGC-1alpha. EMBO Journal.

[45] Canto C., Gerhart-Hines Z., Feige J.N., Lagouge M., Noriega L., Milne J.C. (2009). AMPK regulates energy expenditure by modulating NAD+ metabolism and SIRT1 activity. Nature.

[46] Canto C., Jiang L.Q., Deshmukh A.S., Mataki C., Coste A., Lagouge M. (2010). Interdependence of AMPK and SIRT1 for metabolic adaptation to fasting and exercise in skeletal muscle. Cell Metabolism.

[47] Nin V., Escande C., Chini C.C., Giri S., Camacho-Pereira J., Matalonga J. (2012). Role of deleted in breast cancer 1 (DBC1) protein in SIRT1 deacetylase activation induced by protein kinase A and AMP-activated protein kinase. Journal of Biological Chemistry.

[48] Greer E.L., Oskoui P.R., Banko M.R., Maniar J.M., Gygi M.P., Gygi S.P. (2007). The energy sensor AMP-activated protein kinase directly regulates the mammalian FOXO3 transcription factor. Journal of Biological Chemistry.

[49] Hou X., Xu S., Maitland-Toolan K.A., Sato K., Jiang B., Ido Y. (2008). SIRT1 regulates hepatocyte lipid metabolism through activating AMP-activated protein kinase. Journal of Biological Chemistry.

[50] Sakamoto K., McCarthy A., Smith D., Green K.A., Grahame Hardie D., Ashworth A. (2005). Deficiency of LKB1 in skeletal muscle prevents AMPK activation and glucose uptake during contraction. EMBO Journal.

[51] Lan F., Cacicedo J.M., Ruderman N., Ido Y. (2008). SIRT1 modulation of the acetylation status, cytosolic localization, and activity of LKB1. Possible role in AMP-activated protein kinase activation. Journal of Biological Chemistry.

[52] Philp A., Chen A., Lan D., Meyer G.A., Murphy A.N., Knapp A.E. (2011). Sirtuin 1 (SIRT1) deacetylase activity is not required for mitochondrial biogenesis or peroxisome proliferator-activated receptor-gamma coactivator-1alpha (PGC-1alpha) deacetylation following endurance exercise. Journal of Biological Chemistry.

[53] Dasgupta B., Milbrandt J. (2007). Resveratrol stimulates AMP kinase activity in neurons. Proceedings of the National Academy of Sciences of the United States of America.

[54] Ruderman N.B., Carling D., Prentki M., Cacicedo J.M. (2013). AMPK, insulin resistance, and the metabolic syndrome. Journal of Clinical Investigation.

[55] Ruderman N.B., Xu X.J., Nelson L., Cacicedo J.M., Saha A.K., Lan F. (2010). AMPK and SIRT1: a long-standing partnership?. American Journal of Physiology – Endocrinology and Metabolism.

[56] Gurd B.J., Yoshida Y., Lally J., Holloway G.P., Bonen A. (2009). The deacetylase enzyme SIRT1 is not associated with oxidative capacity in rat heart and skeletal muscle and its overexpression reduces mitochondrial biogenesis. Journal of Physiology.

[57] Sun C., Zhang F., Ge X., Yan T., Chen X., Shi X. (2007). SIRT1 improves insulin sensitivity under insulin-resistant conditions by repressing PTP1B. Cell Metabolism.

[58] Rosen E.D., Spiegelman B.M. (2006). Adipocytes as regulators of energy balance and glucose homeostasis. Nature.

[59] Picard F., Kurtev M., Chung N., Topark-Ngarm A., Senawong T., Oliveira R.Machado De (2004). Sirt1 promotes fat mobilization in white adipocytes by repressing PPAR-gamma. Nature.

[60] Qiang L., Wang L., Kon N., Zhao W., Lee S., Zhang Y. (2012). Brown remodeling of white adipose tissue by SirT1-dependent deacetylation of ppargamma. Cell.

[61] Chalkiadaki A., Guarente L. (2012). High-fat diet triggers inflammation-induced cleavage of SIRT1 in adipose tissue to promote metabolic dysfunction. Cell Metabolism.

[62] Costa Cdos S., Hammes T.O., Rohden F., Margis R., Bortolotto J.W., Padoin A.V. (2010). SIRT1 transcription is decreased in visceral adipose tissue of morbidly obese patients with severe hepatic steatosis. Obesity Surgery.

[63] Gillum M.P., Kotas M.E., Erion D.M., Kursawe R., Chatterjee P., Nead K.T. (2011). SirT1 regulates adipose tissue inflammation. Diabetes.

[64] Xu C., Bai B., Fan P., Cai Y., Huang B., Law I.K. (2013). Selective overexpression of human SIRT1 in adipose tissue enhances energy homeostasis and prevents the deterioration of insulin sensitivity with ageing in mice. American Journal of Translational Research.

[65] Mullican S.E., Tomaru T., Gaddis C.A., Peed L.C., Sundaram A., Lazar M.A. (2013). A novel adipose-specific gene deletion model demonstrates potential pitfalls of existing methods. Journal of Molecular Endocrinology.

[66] Lee K.Y., Russell S.J., Ussar S., Boucher J., Vernochet C., Mori M.A. (2013). Lessons on conditional gene targeting in mouse adipose tissue. Diabetes.

[67] Yoshizaki T., Milne J.C., Imamura T., Schenk S., Sonoda N., Babendure J.L. (2009). SIRT1 exerts anti-inflammatory effects and improves insulin sensitivity in adipocytes. Molecular and Cellular Biology.

[68] Timmons J.A., Wennmalm K., Larsson O., Walden T.B., Lassmann T., Petrovic N. (2007). Myogenic gene expression signature establishes that brown and white adipocytes originate from distinct cell lineages. Proceedings of the National Academy of Sciences of the United States of America.

[69] Wu J., Bostrom P., Sparks L.M., Ye L., Choi J.H., Giang A.H. (2012). Beige adipocytes are a distinct type of thermogenic fat cell in mouse and human. Cell.

[70] Tiraby C., Tavernier G., Lefort C., Larrouy D., Bouillaud F., Ricquier D. (2003). Acquirement of brown fat cell features by human white adipocytes. Journal of Biological Chemistry.

[71] Orci L., Cook W.S., Ravazzola M., Wang M.Y., Park B.H., Montesano R. (2004). Rapid transformation of white adipocytes into fat-oxidizing machines. Proceedings of the National Academy of Sciences of the United States of America.

[72] Puigserver P., Wu Z., Park C.W., Graves R., Wright M., Spiegelman B.M. (1998). A cold-inducible coactivator of nuclear receptors linked to adaptive thermogenesis. Cell.

[73] Seale P., Kajimura S., Yang W., Chin S., Rohas L.M., Uldry M. (2007). Transcriptional control of brown fat determination by PRDM16. Cell Metabolism.

[74] Rosenwald M., Perdikari A., Rulicke T., Wolfrum C. (2013). Bi-directional interconversion of brite and white adipocytes. Nature Cell Biology.

[75] Moynihan K.A., Grimm A.A., Plueger M.M., Bernal-Mizrachi E., Ford E., Cras-Meneur C. (2005). Increased dosage of mammalian Sir2 in pancreatic beta cells enhances glucose-stimulated insulin secretion in mice. Cell Metabolism.

[76] Bordone L., Motta M.C., Picard F., Robinson A., Jhala U.S., Apfeld J. (2006). Sirt1 regulates insulin secretion by repressing UCP2 in pancreatic beta cells. PLoS Biology.

[77] Ramsey K.M., Mills K.F., Satoh A., Imai S. (2008). Age-associated loss of Sirt1-mediated enhancement of glucose-stimulated insulin secretion in beta cell-specific Sirt1-overexpressing (BESTO) mice. Aging Cell.

[78] Wu L., Zhou L., Lu Y., Zhang J., Jian F., Liu Y. (2012). Activation of SIRT1 protects pancreatic beta-cells against palmitate-induced dysfunction. Biochimica et Biophysica Acta.

[79] Luu L., Dai F.F., Prentice K.J., Huang X., Hardy A.B., Hansen J.B. (2013). The loss of Sirt1 in mouse pancreatic beta cells impairs insulin secretion by disrupting glucose sensing. Diabetologia.

[80] Bai P., Canto C., Brunyanszki A., Huber A., Szanto M., Cen Y. (2011). PARP-2 regulates SIRT1 expression and whole-body energy expenditure. Cell Metabolism.

[81] Kitamura T., Nakae J., Kitamura Y., Kido Y., Biggs W.H., Wright C.V. (2002). The forkhead transcription factor Foxo1 links insulin signaling to Pdx1 regulation of pancreatic beta cell growth. Journal of Clinical Investigation.

[82] Bastien-Dionne P.O., Valenti L., Kon N., Gu W., Buteau J. (2011). Glucagon-like peptide 1 inhibits the sirtuin deacetylase SirT1 to stimulate pancreatic beta-cell mass expansion. Diabetes.

[83] Lee J.H., Song M.Y., Song E.K., Kim E.K., Moon W.S., Han M.K. (2009). Overexpression of SIRT1 protects pancreatic beta-cells against cytokine toxicity by suppressing the nuclear factor-kappaB signaling pathway. Diabetes.

[84] Gao Q., Horvath T.L. (2008). Neuronal control of energy homeostasis. FEBS Letters.

[85] Ramadori G., Lee C.E., Bookout A.L., Lee S., Williams K.W., Anderson J. (2008). Brain SIRT1: anatomical distribution and regulation by energy availability. Journal of Neuroscience.

[86] Satoh A., Brace C.S., Ben-Josef G., West T., Wozniak D.F., Holtzman D.M. (2010). SIRT1 promotes the central adaptive response to diet restriction through activation of the dorsomedial and lateral nuclei of the hypothalamus. Journal of Neuroscience.

[87] Dietrich M.O., Antunes C., Geliang G., Liu Z.W., Borok E., Nie Y. (2010). Agrp neurons mediate Sirt1's action on the melanocortin system and energy balance: roles for Sirt1 in neuronal firing and synaptic plasticity. Journal of Neuroscience.

[88] Ramadori G., Fujikawa T., Fukuda M., Anderson J., Morgan D.A., Mostoslavsky R. (2010). SIRT1 deacetylase in POMC neurons is required for homeostatic defenses against diet-induced obesity. Cell Metabolism.

[89] Cohen D.E., Supinski A.M., Bonkowski M.S., Donmez G., Guarente L.P. (2009). Neuronal SIRT1 regulates endocrine and behavioral responses to calorie restriction. Genes Development.

[90] Satoh A., Brace C.S., Rensing N., Cliften P., Wozniak D.F., Herzog E.D. (2013). Sirt1 extends life span and delays aging in mice through the regulation of Nk2 Homeobox 1 in the DMH and LH. Cell Metabolism.

[91] Cakir I., Perello M., Lansari O., Messier N.J., Vaslet C.A., Nillni E.A. (2009). Hypothalamic Sirt1 regulates food intake in a rodent model system. PLoS One.

[92] Sasaki T., Kim H.J., Kobayashi M., Kitamura Y.I., Yokota-Hashimoto H., Shiuchi T. (2010). Induction of hypothalamic Sirt1 leads to cessation of feeding via agouti-related peptide. Endocrinology.

[93] Chang H.C., Guarente L. (2013). SIRT1 mediates central circadian control in the SCN by a mechanism that decays with aging. Cell.

[94] Pacholec M., Bleasdale J.E., Chrunyk B., Cunningham D., Flynn D., Garofalo R.S. (2010). SRT1720, SRT2183, SRT1460, and resveratrol are not direct activators of SIRT1. Journal of Biological Chemistry.

[95] Hubbard B.P., Gomes A.P., Dai H., Li J., Case A.W., Considine T. (2013). Evidence for a common mechanism of SIRT1 regulation by allosteric activators. Science.

[96] Howitz K.T., Bitterman K.J., Cohen H.Y., Lamming D.W., Lavu S., Wood J.G. (2003). Small molecule activators of sirtuins extend Saccharomyces cerevisiae lifespan. Nature.

[97] Milne J.C., Lambert P.D., Schenk S., Carney D.P., Smith J.J., Gagne D.J. (2007). Small molecule activators of SIRT1 as therapeutics for the treatment of type 2 diabetes. Nature.

[98] Lagouge M., Argmann C., Gerhart-Hines Z., Meziane H., Lerin C., Daussin F. (2006). Resveratrol improves mitochondrial function and protects against metabolic disease by activating SIRT1 and PGC-1alpha. Cell.

[99] Feige J.N., Lagouge M., Canto C., Strehle A., Houten S.M., Milne J.C. (2008). Specific SIRT1 activation mimics low energy levels and protects against diet-induced metabolic disorders by enhancing fat oxidation. Cell Metabolism.

[100] Park S.J., Ahmad F., Philp A., Baar K., Williams T., Luo H. (2012). Resveratrol ameliorates aging-related metabolic phenotypes by inhibiting cAMP phosphodiesterases. Cell.

[101] Harikumar K.B., Aggarwal B.B. (2008). Resveratrol: a multitargeted agent for age-associated chronic diseases. Cell Cycle.

[102] Gledhill J.R., Montgomery M.G., Leslie A.G., Walker J.E. (2007). Mechanism of inhibition of bovine F1-ATPase by resveratrol and related polyphenols. Proceedings of the National Academy of Sciences of the United States of America.

[103] Hawley S.A., Ross F.A., Chevtzoff C., Green K.A., Evans A., Fogarty S. (2010). Use of cells expressing gamma subunit variants to identify diverse mechanisms of AMPK activation. Cell Metabolism.

[104] Um J.H., Park S.J., Kang H., Yang S., Foretz M., McBurney M.W. (2010). AMP-activated protein kinase-deficient mice are resistant to the metabolic effects of resveratrol. Diabetes.

[105] Narkar V.A., Downes M., Yu R.T., Embler E., Wang Y.X., Banayo E. (2008). AMPK and PPARdelta agonists are exercise mimetics. Cell.

[106] Long Y.C., Zierath J.R. (2006). AMP-activated protein kinase signaling in metabolic regulation. Journal of Clinical Investigation.

[107] Zhao W., Kruse J.P., Tang Y., Jung S.Y., Qin J., Gu W. (2008). Negative regulation of the deacetylase SIRT1 by DBC1. Nature.

[108] Kim J.E., Chen J., Lou Z. (2008). DBC1 is a negative regulator of SIRT1. Nature.

[109] Escande C., Chini C.C., Nin V., Dykhouse K.M., Novak C.M., Levine J. (2010). Deleted in breast cancer-1 regulates SIRT1 activity and contributes to high-fat diet-induced liver steatosis in mice. Journal of Clinical Investigation.

[110] Yuan J., Luo K., Liu T., Lou Z. (2012). Regulation of SIRT1 activity by genotoxic stress. Genes Development.

[111] Zannini L., Buscemi G., Kim J.E., Fontanella E., Delia D. (2012). DBC1 phosphorylation by ATM/ATR inhibits SIRT1 deacetylase in response to DNA damage. Journal of Molecular Cell Biology.

[112] Kim E.J., Kho J.H., Kang M.R., Um S.J. (2007). Active regulator of SIRT1 cooperates with SIRT1 and facilitates suppression of p53 activity. Molecular Cell.

[113] Lakshminarasimhan M., Curth U., Moniot S., Mosalaganti S., Raunser S., Steegborn C. (2013). Molecular architecture of the human protein deacetylase Sirt1 and its regulation by AROS and resveratrol. Bioscience Reports.

[114] Bai P., Canto C. (2012). The role of PARP-1 and PARP-2 enzymes in metabolic regulation and disease. Cell Metabolism.

[115] Bai P., Canto C., Oudart H., Brunyanszki A., Cen Y., Thomas C. (2011). PARP-1 inhibition increases mitochondrial metabolism through SIRT1 activation. Cell Metabolism.

[116] Aksoy P., Escande C., White T.A., Thompson M., Soares S., Benech J.C. (2006). Regulation of SIRT 1 mediated NAD dependent deacetylation: a novel role for the multifunctional enzyme CD38. Biochemical and Biophysical Research Communications.

[117] Barbosa M.T., Soares S.M., Novak C.M., Sinclair D., Levine J.A., Aksoy P. (2007). The enzyme CD38 (a NAD glycohydrolase, EC 3.2.2.5) is necessary for the development of diet-induced obesity. FASEB Journal.

[118] Yoshino J., Mills K.F., Yoon M.J., Imai S. (2011). Nicotinamide mononucleotide, a key NAD(+) intermediate, treats the pathophysiology of diet- and age-induced diabetes in mice. Cell Metabolism.

[119] Caton P.W., Kieswich J., Yaqoob M.M., Holness M.J., Sugden M.C. (2011). Nicotinamide mononucleotide protects against pro-inflammatory cytokine-mediated impairment of mouse islet function. Diabetologia.

[120] Canto C., Houtkooper R.H., Pirinen E., Youn D.Y., Oosterveer M.H., Cen Y. (2012). The NAD(+) precursor nicotinamide riboside enhances oxidative metabolism and protects against high-fat diet-induced obesity. Cell Metaboslim.

[121] Araki T., Sasaki Y., Milbrandt J. (2004). Increased nuclear NAD biosynthesis and SIRT1 activation prevent axonal degeneration. Science.

[122] Revollo J.R., Grimm A.A., Imai S. (2004). The NAD biosynthesis pathway mediated by nicotinamide phosphoribosyltransferase regulates Sir2 activity in mammalian cells. Journal of Biological Chemistry.

[123] Zhang T., Berrocal J.G., Frizzell K.M., Gamble M.J., DuMond M.E., Krishnakumar R. (2009). Enzymes in the NAD+ salvage pathway regulate SIRT1 activity at target gene promoters. Journal of Biological Chemistry.

[124] Wu J., Zhang F., Yan M., Wu D., Yu Q., Zhang Y. (2011). WldS enhances insulin transcription and secretion via a SIRT1-dependent pathway and improves glucose homeostasis. Diabetes.

[125] Schmeisser K., Mansfeld J., Kuhlow D., Weimer S., Priebe S., Heiland I. (2013). Role of sirtuins in lifespan regulation is linked to methylation of nicotinamide. Nature Chemical Biology.

[126] Feldman J.L., Baeza J., Denu J.M. (2013). Activation of the protein deacetylase SIRT6 by long-chain fatty acids and widespread deacylation by mammalian sirtuins. Journal of Biological Chemistry.

[127] Mouchiroud L., Houtkooper R.H., Moullan N., Katsyuba E., Ryu D., Canto C. (2013). The NAD(+)/sirtuin pathway modulates longevity through activation of mitochondrial UPR and FOXO signaling. Cell.

[128] Hsu C.P., Zhai P., Yamamoto T., Maejima Y., Matsushima S., Hariharan N. (2010). Silent information regulator 1 protects the heart from ischemia/reperfusion. Circulation.

[129] Alcendor R.R., Gao S., Zhai P., Zablocki D., Holle E., Yu X. (2007). Sirt1 regulates aging and resistance to oxidative stress in the heart. Circulation Research.

[130] Oka S., Alcendor R., Zhai P., Park J.Y., Shao D., Cho J. (2011). PPARalpha-Sirt1 complex mediates cardiac hypertrophy and failure through suppression of the ERR transcriptional pathway. Cell Metabolism.

[131] Kawashima T., Inuzuka Y., Okuda J., Kato T., Niizuma S., Tamaki Y. (2011). Constitutive SIRT1 overexpression impairs mitochondria and reduces cardiac function in mice. Journal of Molecular and Cellular Cardiology.

